# All-oral combination of oral vinorelbine and capecitabine as first-line chemotherapy in HER2-negative metastatic breast cancer: an International Phase II Trial

**DOI:** 10.1038/sj.bjc.6605156

**Published:** 2009-07-07

**Authors:** N Tubiana-Mathieu, P Bougnoux, D Becquart, A Chan, P-F Conte, F Majois, M Espie, M Morand, N Vaissiere, G Villanova

**Affiliations:** 1 CHU Dupuytren, Limoges, France; 2 CHU Bretonneau, Tours, France; 3 AZ Middelheim, Antwerp, Belgium; 4 Mount Hospital and Royal Perth Hospital, Perth, Australia; 5 Policlinico di Modena, Modena, Italy; 6 Hopital Jolimont, Haine St Paul, Belgium; 7 Hopital St Louis, Paris, France; 8 Institut de Recherche Pierre Fabre, Boulogne-Billancourt, France

**Keywords:** oral therapy, first-line chemotherapy, metastatic breast cancer, HER2 negative

## Abstract

**Background:**

This multicentre, international phase II trial evaluated the efficacy and safety profile of a first-line combination of oral vinorelbine plus capecitabine for women with metastatic breast cancer (MBC).

**Methods:**

Patients with measurable, HER2-negative disease received, as a first line in metastatic setting, 3-weekly cycles of oral vinorelbine 80 mg m^−2^ (after a first cycle at 60) on day 1 and day 8, plus capecitabine 1000 mg m^−2^ (750 if ⩾65 years of age) twice daily, on days 1–14. Treatment was continued until progression or unacceptable toxicity.

**Results:**

A total of 55 patients were enrolled and 54 were treated (median age: 58.5 years). Most (78%) had visceral involvement and 63% had received earlier (neo)adjuvant chemotherapy. The objective response rate (RECIST) in 49 evaluable patients was 51% (95% confidence interval (CI), 36–66), including complete response in 4%. The clinical benefit rate (response or stable disease for ⩾6 months) was 63% (95% CI, 48–77). The median duration of response was 7.2 months (95% CI, 6.4–10.2). After a median follow-up of 41 months, median progression-free survival was 8.4 months (95% CI, 5.8–9.7) and median overall survival was 29.2 months (95% CI, 18.2–40.1). Treatment-related adverse events were manageable, the main grade 3–4 toxicity was neutropaenia (49%); two patients experienced febrile neutropaenia and three patients had a neutropaenic infection (including one septic death). A particularly low rate of alopaecia was observed.

**Conclusion:**

These results show that the all-oral combination of oral vinorelbine and capecitabine is an effective and well-tolerated first-line regimen for MBC.

Breast cancer is among the most common cancers in Western countries. Advanced breast cancer is the leading cause of death in women aged from 40 to 54 years. About one in eight Western women will develop breast cancer if they live up to an age of 85 years. Despite improving locoregional and adjuvant treatment, many patients still develop recurrent and/or metastatic breast cancer (MBC) within 10 years and will subsequently die of the disease (Harris *et al*, 2000).

There is no single standard of care for patients with MBC, as treatment plans require an individualised approach based on multiple factors, including tumour biology, growth rate of disease, presence of visceral metastases, history of earlier therapy and response, risk for toxicity and patient preference (O’Shaughnessy, 2005). Preferred first-line single agents in advanced breast cancer are anthracyclines, taxanes, capecitabine, gemcitabine and vinorelbine (NCCN Guidelines, 2009). More recently, bevacizumab has been evaluated in combination with taxanes with interesting clinical results. With anthracyclines and taxanes being used increasingly in early-stage setting, there is an even greater need for other active options in advanced setting to improve outcomes and/or quality of life. The development of oral chemotherapy formulations should allow a higher efficiency, by providing consistent efficacy and reduced patient constraints.

A phase II trial has evaluated oral vinorelbine (Navelbine Oral) single agent as first-line chemotherapy for locally advanced or metastatic breast cancer, showing an overall response rate of 31% and a median progression-free survival (PFS) of 17.4 weeks (Freyer *et al*, 2003). Capecitabine (Xeloda) as a single agent has been largely studied in pre-treated metastatic patients, with a median response rate of 28% and a median time to progression of 4.7 months (Ershler, 2006). Both agents have been included among commonly used compounds for the treatment of MBC in ESMO Clinical Recommendations (Kataja and Castiglione, 2008).

After establishing both oral vinorelbine and capecitabine as a standard of care in MBC, the development of the combination of both agents has a very strong rationale. Moreover, the two agents have different mechanisms of action, different and acceptable safety profiles and synergistic antitumour activity in preclinical models (Sawada *et al*, 2002). The combination of intravenous vinorelbine (Navelbine) and capecitabine has shown promising efficacy in MBC (Hess *et al*, 2004; Ghosn *et al*, 2006; Iodice *et al*, 2006; Welt *et al*, 2006; Palumbo *et al*, 2008). By combining oral vinorelbine and capecitabine, similar outcomes might be achieved without the burden of intravenous infusion.

Three phase I trials have evaluated the combination of oral vinorelbine and capecitabine in MBC (Kellokumpu-Lehtinen *et al*, 2006; Nole *et al*, 2006; Anton *et al*, 2008). In all these studies, the recommended dose of capecitabine for phase II trials was 1000 mg m^−2^ twice daily, on days 1–14. As special caution is needed with capecitabine in combination for elderly patients, the doses administered in this trial were reduced to 750 mg m^−2^ twice daily, on days 1–14 if age was ⩾65 years. Regarding oral vinorelbine, different schedules have been evaluated and, among these options, we evaluated in our trial the schedule of 60 mg m^−2^ on days 1 and 8 for cycle 1, with a dose escalation to 80 mg m^−2^ for subsequent cycles in the absence of grade 3 or 4 haematological toxicity.

This international, open-label, phase II trial was designed to evaluate the activity and safety of an all-oral combination of oral vinorelbine and capecitabine as first-line therapy for patients with HER2-negative MBC.

## Materials and methods

### Eligibility criteria

Eligible patients were female, ⩾18 years, with documented metastatic breast adenocarcinoma untreated by chemotherapy. Other inclusion criteria included HER2-negative disease (IHC 0–1 or IHC 2+ confirmed as FISH negative), Karnofsky performance status ⩾70%, at least one measurable lesion according to RECIST criteria (Therasse *et al*, 2000) and a life expectancy ⩾16 weeks. Adjuvant or neoadjuvant chemotherapy containing an anthracycline and/or a taxane was allowed if ⩾6 months had elapsed between the last dose of chemotherapy and documentation of relapse. Earlier hormone therapy for advanced disease was allowed. Patients were required to have adequate bone marrow and hepatic and renal functions, indicated by haemoglobin ⩾10 g per 100 ml, absolute neutrophil count ⩾2 × 10^9^ per l, platelet count ⩾100 × 10^9^ per l, total serum bilirubin ⩽1.5 × upper normal limit (UNL), AST/ALT ⩽2.5 × UNL, (⩽3.5 × UNL in case of liver metastases), alkaline phosphatase ⩽2.5 UNL (or ⩽5 UNL for bone metastases) and creatinine clearance >50 ml min^−1^ (calculated using the Cockroft and Gault formula). Patients were required to give written informed consent before study-specific procedures were performed and to comply with protocol for the duration of the study.

Patients were ineligible if they had only local relapse, earlier chemotherapy in a metastatic setting, previous exposure to a vinca-alkaloid or capecitabine, serious illness or medical conditions such as cardiac disease, unstable diabetes, uncontrolled hypercalcaemia, severe peripheral neuropathy, active infection or previous organ allograft. Patients were also excluded if they were pregnant or lactating, required a concurrent use of the antiviral sorivudine or a chemically related analogue such as brivudine, had clinical central nervous system (CNS) or leptomeningeal metastases, had a malabsorption disease that may affect absorption or oral chemotherapy, had possible hypersensitivity to fluoropyrimidine therapy, had participated in another clinical trial with any investigational drug within 30 days before study inclusion or had a history of another malignancy except cured basal-cell carcinoma of the skin or excised carcinoma *in situ* of the cervix.

### Primary and secondary end points

The primary end point of the study was overall response. Overall response was defined as the best confirmed response recorded from the date of registration until the end of study period. Secondary objectives included the evaluation of safety, duration of response, PFS and overall survival.

### Treatment plan

Treatment was provided in 3-weekly cycles. Oral vinorelbine was administered at 60 mg m^−2^ on days 1 and 8 of the first cycle and escalated to 80 mg m^−2^ at cycle 2 and subsequent cycles in the absence of grade 3 or 4 haematological toxicity.

Capecitabine was administered at a dose of 1000 mg m^−2^ twice daily, on days 1–14 (750 mg m^−2^ twice daily for patients ⩾65 years).

Prophylactic oral antiemetic medication with a 5-HT_3_ antagonist was recommended before each oral vinorelbine administration.

Treatment was continued until disease progression, unacceptable toxicity or patient's refusal.

### Dose modifications

Dose adjustments and/or treatment delays could be made in the event of dose-limiting haematological or non-haematological toxicities. If study treatment could not be administered after two delays (meaning 2 weeks) of the theoretical day 1 because of any toxicity, it had to be permanently discontinued. Thus, the maximum interval between the start of one cycle and the next was 5 weeks. If one of the agents had to be permanently discontinued, the patient was withdrawn from the study.

Oral vinorelbine was not administered if patients had grade ⩾2 neutropaenia, and capecitabine was interrupted in case of grade ⩾3 neutropaenia. After one episode of grade 3 or 4 neutropaenia, the dose of oral vinorelbine was permanently decreased to 60 mg m^−2^ for subsequent cycles. Patients experiencing grade 3 or 4 neutropaenia, with or without fever, were allowed to receive a granulocyte colony-stimulating factor (G-CSF) in subsequent cycles at the investigator's discretion. If AST/ALT/alkaline phosphatase increased to >5.0 UNL, or if bilirubin increased to >1.5 UNL, both agents were not administered and a reassessment was carried out a week later. If grade ⩾2 diarrhoea or hand–foot syndrome occurred, administration of capecitabine was interrupted until it resolved to grade 0 or 1 and doses were decreased by 25% (if grade 3) or 50% (if grade 4) for subsequent cycles.

### Study assessments

Every patient who entered the study underwent baseline assessments, including medical history, physical examination, performance status, HER2 testing (either on the primary tumour or on a metastatic site), electrocardiogram and chest X-ray. Tumour measurements by imaging were determined as clinically indicated by computed tomography (CT), abdominal ultrasound, bone scan and/or brain CT scan (if suspicion of CNS involvement). Complete blood cell counts were carried out within 2 days before each oral vinorelbine administration.

Responses were assessed every two cycles until disease progression, or more frequently if early progression was suspected. The best overall response achieved, according to RECIST criteria, was reported for each patient. A complete response (CR) required a complete disappearance of all lesions, and a partial response (PR) required at least a 30% decrease of the sum of the longest diameters of target lesions. Both CR and PR had to be confirmed at least 4 weeks later. Stable disease (SD) was defined as neither sufficient shrinkage to qualify for PR nor sufficient increase to qualify for progressive disease (PD). Progressive disease was defined as at least a 20% increase in the sum of longest diameters of target lesions and/or the appearance of new lesions. Clinical benefit was defined as patients achieving CR, PR or SD, maintained for a minimum of 6 months.

Adverse events and medical history were recorded throughout the study. The severity of adverse events was graded according to NCI common toxicity criteria version 2.0.

### Statistical analysis

The one-sample multiple testing procedure for phase II clinical trials as described by Fleming (1982) was used. This procedure uses the standard single-stage test procedure at the last one of two pre-specified tests, while allowing for early termination (should extreme results be seen) and essentially preserving the size and power of the single-stage procedure.

On the basis of a type I error rate of 5% and a 95% power to reject the null hypothesis of a 25% objective response rate (complete or partial), a sample size of 55 patients was needed.

All treated patients were included in the intent-to-treat (ITT) analysis and were analysed for safety. The evaluable population was defined as all patients eligible for the trial who underwent a full evaluation of target and non-target lesions and had received at least two cycles of study treatment (including patients with PD documented before the second cycle).

Response rate and clinical benefit were tabulated together with 95% confidence interval (CI), following the exact method. The Kaplan–Meier method was applied to overall survival, PFS and duration of response. Subset analysis (according to baseline characteristics) was performed for response rate.

## Results

### Patient characteristics

Between March 2004 and June 2005, 55 patients with MBC were enrolled from 13 sites in six countries. One patient who entered into the study did not receive study treatment because of the presence of a major exclusion criterion (low creatinine clearance). Five of the remaining 54 patients were not evaluable for response, but were included in the ITT analysis: one patient was not eligible for the study (no measurable disease as defined in the protocol), two patients were not assessable as a result of premature study discontinuation (one patient withdrew from the study after the first therapy administration owing to abdominal pain, the second patient died during the second cycle from sepsis) and two patients having received three and six cycles did not undergo a full evaluation of all target and non-target lesions. Therefore, 49 patients with measurable disease were assessable for disease response per protocol.

The patients’ characteristics are described in Table 1. Patients typically had visceral metastases and a good performance status. All had HER2-negative disease. Sixty-three percent of patients had received earlier (neo)adjuvant chemotherapy, including an anthracycline in the majority and a taxane in some cases.

### Clinical efficacy

The objective response rate was 51% among the 49 evaluable patients (95% CI, 36.3–65.6), including CRs in 4.1%. Clinical benefit (CR+PR+SD ⩾6 months) was 63.3% (95% CI, 48.3–76.6). In the ITT population, the objective response rate was 46.3% (95% CI, 32.6–60.4). Median time to response was 3.1 months (range 1.3–6.7) and median duration of response was 7.2 months (95% CI, 6.4–10.2) (Table 2).

A subanalysis of responses according to patients’ characteristics is shown in Table 3. It is noteworthy that the response rate in patients with liver involvement (52.2%) was similar to the rate in the overall population.

**Figure 1:**
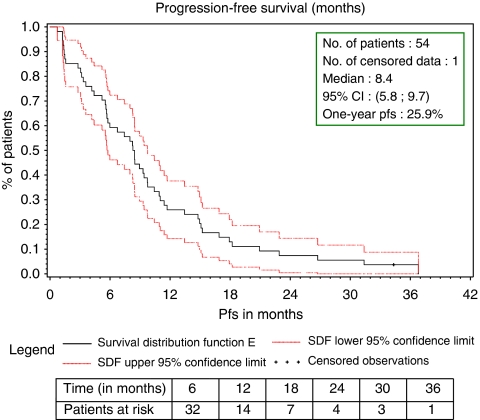
Progression-free survival (months) intent-to-treat analysis.

**Figure 2:**
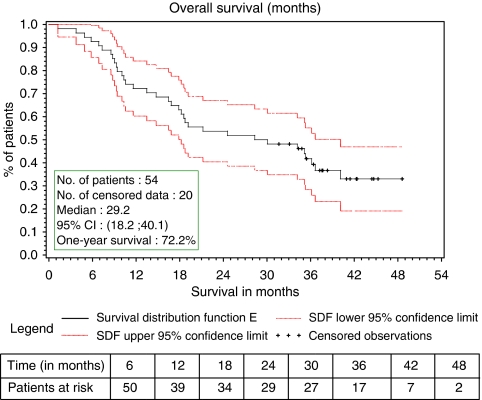
Overall survival (months) intent-to-treat analysis.

After a median follow-up of 41 months, 20 of the 54 patients treated in the study were still alive. Median PFS was 8.4 months (95% CI, 5.8–9.7 – Figure 1). Median PFS in the ER-positive (*n*=39) and ER-negative (*n*=12) population was 8.9 (95% CI, 6.7–11.0) and 4.0 (95% CI, 1.4–8.2) months, respectively. Median overall survival was 29.2 months (95% CI, 18.2–40.1 – Figure 2).

The majority of patients received further lines of treatment after discontinuation of study therapy: chemotherapy (85% of patients), hormone therapy (54%) and radiotherapy (33%).

### Drug delivery

The median number of cycles was seven (range: 1–58). Thirteen patients received study treatment for more than 40 weeks, and eight patients were treated for more than 1 year. The median relative dose intensity of oral vinorelbine and capecitabine was 86.8 and 86.7%, respectively. Dose escalation of oral vinorelbine was achieved in more than 90% of the patients. In 22 patients (41%), the dose of one or both of the agents was reduced. Every oral vinorelbine intake scheduled at day 1 could be administered and only 6.2% of the doses planned at day 8 had to be omitted.

In eight patients (14.8%), G-CSF was administered to manage neutropaenia with a curative intent. Two of these patients also received G-CSF with a preventive purpose. In all, G-CSF was administered in 14 cycles (2.8%).

### Treatment-related toxicity

Table 4 shows the incidence of the most common grade 3–4 side effects related to treatment. The most frequent haematological toxicity was neutropaenia, with grade 3–4 neutropaenia being observed in 49% of patients. Neutropaenia was usually brief and infrequently associated with infections. Only two patients (3.8%) experienced febrile neutropaenia. Three additional patients (5.6%) had documented infection associated with grade 3–4 neutropaenia (one fatal septicaemia, one pneumonia and one urinary tract infection). The septic death occurred in a 65-year-old patient with a medical history of non-insulin-dependent diabetes mellitus and a mitral valve replacement who presented, during the second cycle, a positive blood culture in a context of grade 4 neutropaenia and grade 3 diarrhoea.

Non-haematological toxicities were mild. The main grade 3 gastrointestinal adverse event was vomiting, which was seen in 9.3% of patients and in 1% of cycles. The incidence of grade 3–4 diarrhoea was observed in 3.7% of patients and in 0.6% of cycles without any grade 4. Grade 2 alopaecia was observed in 9.3% and no grade 3–4 neuropathy was observed.

## Discussion

This multicentre study confirms the efficacy of oral vinorelbine with capecitabine previously seen in other studies, with a consistent response rate of more than 50% (Delcambre *et al*, 2005; Finek *et al*, 2009; Nole *et al*, 2009). The response rate in our trial is comparable with the activity observed with intravenous combinations. Moreover, a similar response rate was achieved in the critical segment of patients having visceral metastases. Disease control was obtained in two-thirds of the patients.

Secondary efficacy end points were also very encouraging: median PFS was 8.4 months and median overall survival was 29.2 months.

There is little evidence from trials reported that major differences exist between many commonly used chemotherapy regimens (Wilcken and Dear, 2008). Among them, the combinations of a taxane with an antimetabolite have been evaluated in two major phase III trials (O’Shaughnessy *et al*, 2002; Albain *et al*, 2008). The activity observed with the combination of oral vinorelbine and capecitabine in our trial compares favourably with any of these regimens and provides a strong argument for its development in randomised, comparative trials with any of them.

The results of a randomised phase III trial evaluating, as a first-line treatment for HER2-negative MBC patients, the combination of paclitaxel and bevacizumab *vs* paclitaxel alone have been published recently (Miller *et al*, 2007). The patient populations entered in this study and in our trial present some similar characteristics (around one-third of patients without any previous chemotherapy and about 10% of previous anthracycline and taxane in early-stage setting). There is no major difference in the outcome of patients in terms of overall response rate and median overall survival between our trial and the paclitaxel–bevacizumab regimen. Moreover, the selection of patients who may benefit from a bevacizumab-based regimen is not clear yet, this fact being a critical issue because of its high cost.

The toxicities associated with oral vinorelbine and capecitabine were predictable and manageable. As in other phase II studies evaluating this regimen, significant haematological and non-haematological adverse events were not frequently observed. Nevertheless, as oral chemotherapy is largely taken at home by patients, it is recommended to provide them with adequate information about the measures to be taken in case of gastro-intestinal adverse events and to manage them appropriately. The rate of alopaecia in patients receiving this combination was particularly low.

The good tolerability of the combination is apparent from the long duration of therapy in our study. Chemotherapy could be continued until disease progression, with few patients requiring a cessation of treatment because of intolerable adverse effects. The median number of cycles given was seven, and some patients received treatment for more than 1 year. Two meta-analyses comparing longer *vs* shorter chemotherapy duration in MBC patients have shown that a longer duration was linked with a survival benefit (Coates *et al*, 2003; Gennari *et al*, 2008).

Several surveys have shown that, provided efficacy and tolerability are not compromised, most patients prefer oral to intravenous chemotherapy (Liu *et al*, 1997; Findlay *et al*, 2008). Oral chemotherapy may reduce anxiety in patients who are afraid of injections. Whether it is administered at home or at the hospital, it reduces treatment-related constraints by requiring fewer and shorter hospital visits and has a smaller impact on daily activities (Findlay *et al*, 2008). Therefore, with a similar efficacy and fewer constraints, this all-oral combination could be considered as being more efficient than intravenous treatments.

In conclusion, the oral vinorelbine/capecitabine combination evaluated in this study is effective and well tolerated. This, together with all the benefits of an oral treatment, the possibility of a longer duration of chemotherapy and of a prolonged infusion-free survival, makes it an attractive regimen that could be proposed as a valid option in first line for HER2-negative, MBC patients.

## Figures and Tables

**Table 1:** Patient characteristics

**Characteristics**	***N*=54**	**(%)**
*Median age*	58.5 years	
Range	(31.0–84.0)	
<65	32	59.3
⩾65	22	40.7
		
*Karnofsky performance status at baseline*		
70/80	11	20.4
90/100	43	79.6
		
*Hormonal status*		
ER+/PR+	29	53.6
ER+/PR−	9	16.7
ER−/PR+	2	3.7
ER−/PR−	9	16.7
ER and/or PR unknown	5	9.3
		
Prior chemotherapy (early stage)	34	63.0
		
*Type of Chemotherapy*		
Anthracycline-based without taxane	23	42.6
Anthracycline+taxane	6	11.1
CMF	5	9.3
		
*Prior hormone therapy*	41	75.9
For advanced disease	27	50.0
		
*Number of metastatic sites*		
1	7	13.0
2	22	40.7
>2	25	46.3
		
Visceral involvement	42	77.8
		
*Metastatic sites*		
Liver/lung metastases	26/25	48.1/46.3
Bone metastases	33	61.1
Skin/soft tissue	3/5	5.6/9.3
		
Median delay between diagnosis and first relapse	34.3 months	

**Table 2:** Response to treatment

**Objective response rate (RECIST) Evaluable population**	***n*=49**	**(%)**
CR	2	4.1
PR	23	46.9
Objective response – CR+ PR − (95% CI)	25	51.0 (36.3–65.6)
SD	14	28.6
PD	10	20.4
Clinical benefit (CR+PR+SD ⩾6 months) (95% CI)	31	63.3 (48.3–76.6)
Median time to response (range)	3.1 months (1.3–6.7)	
Median duration of response (95% CI)	7.2 months (6.4–10.2)	
		

CI=confidence interval; CR=complete response; PR=partial response; SD=stable disease.

**Table 3:** Subanalysis of responses according to patients’ characteristics

**Overall response rate (RECIST)**	**(%)**
All evaluable patients (*n*=49)	51.0
Liver metastases (*n*=23)	52.2
No prior chemotherapy (*n*=16)	68.8
Prior anthracycline-based chemotherapy without taxane (*n*=23)	39.1
Prior anthracycline+taxane (*n*=5)	40.0
Prior CMF (*n*=5)	60.0
Prior hormone therapy (*n*=36)	55.6
Prior hormone therapy for MBC (*n*=25)	56.0
Triple-negative disease (*n*=9)	22.2

CMF=cyclophosphamide, methotrexate, 5-FU; MBC=metastatic breast cancer.

**Table 4:** Treatment-related adverse events

	**Per patient (%) *N*=53^a^**		**Per cycle (%) *N*=496**	
**Adverse events by NCI/CTC v.2.0**	**Grade 3**	**Grade 4**	**Grade 3**	**Grade 4**
Anaemia	1.9	1.9	0.2	0.2
Leukopaenia	17.0	11.3	3.6	1.4
Neutropaenia	26.4	22.6	6.5	3.0
Thrombocytopaenia	1.9	0	0.2	0
Febrile neutropaenia	3.8		0.4	
			
	**Per patient (%) *N*=54**		**Per cycle (%) *N*=499**	
	**Grade 3**	**Grade 4**	**Grade 3**	**Grade 4**
Nausea	3.7	0	0.4	0
Vomiting	9.3	0	1.0	0
Diarrhoea	3.7	0	0.6	0
Stomatitis	5.6	1.9	0.6	0.2
Hand–foot syndrome	3.7	0	0.8	0
Fatigue	7.4	0	1.2	0
Infection with G3/4 neutropaenia	3.7	1.9	0.6	0.2
Infection without G3/4 neutropaenia	3.7	0	0.4	0
Thrombosis/embolism	1.9	1.9	0.2	0.2
Anorexia	1.9	0	0.4	0
				
	**Grade 1**	**Grade 2**		
Alopaecia	16.7	9.3		

a One patient was not evaluable for haematological adverse events.
